# Efficacy and Safety of Peroral Endoscopic Myotomy for Esophageal Achalasia and Achalasia-Related Diseases in Patients Aged 75 Years and Over

**DOI:** 10.3390/healthcare9121668

**Published:** 2021-12-01

**Authors:** Jun Nakamura, Takuto Hikichi, Minami Hashimoto, Mika Takasumi, Tsunetaka Kato, Ryoichiro Kobashi, Takumi Yanagita, Rei Suzuki, Mitsuru Sugimoto, Yuki Sato, Hiroki Irie, Tadayuki Takagi, Masao Kobayakawa, Hiromasa Ohira

**Affiliations:** 1 Department of Endoscopy, Fukushima Medical University Hospital, Fukushima 960-1295, Japan; junn7971@fmu.ac.jp (J.N.); mi-hashi@fmu.ac.jp (M.H.); tsune-k@fmu.ac.jp (T.K.); rkobashi@fmu.ac.jp (R.K.); mkobaya@fmu.ac.jp (M.K.); 2 Department of Gastroenterology, Fukushima Medical University School of Medicine, Fukushima 960-1295, Japan; paper@fmu.ac.jp (M.T.); takumi-y@fmu.ac.jp (T.Y.); subaru@fmu.ac.jp (R.S.); kita335@fmu.ac.jp (M.S.); dorcus@fmu.ac.jp (Y.S.); hirokiri@fmu.ac.jp (H.I.); daccho@fmu.ac.jp (T.T.); h-ohira@fmu.ac.jp (H.O.); 3 Medical Research Center, Fukushima Medical University, Fukushima 960-1295, Japan

**Keywords:** elderly people, esophageal achalasia, gastroesophageal reflux disease, jackhammer esophagus, peroral endoscopic myotomy

## Abstract

Peroral endoscopic myotomy (POEM) has become a popular treatment for esophageal achalasia and other esophageal motility disorders. However, its efficacy and safety in elderly patients are unclear. To clarify that, we reviewed the medical records of patients who underwent POEM in our hospital. A total of 11 patients who underwent POEM for esophageal achalasia (*n* = 10) and jackhammer esophagus (*n* = 1) were included. Procedural success, defined as the completion of an esophageal and gastric myotomy, was 100%. Clinical success, defined as an Eckardt score of 3 or less, without the use of additional treatments at 2 months, was 100%. The median Eckardt score significantly decreased after the POEM (baseline vs. 2 months after POEM; 7 (2–8) vs. 0 (0–1), *p* < 0.01). In the second and third years, the cumulative treatment effect maintenance rate was 88.9%. All patients taking antithrombotic agents had safe operations with the temporary discontinuation of these agents. There were four adverse events (two pneumoperitoneum, one mucosal injury, and one pneumonia), all of which improved with fasting or antibiotics. In conclusion, POEM is an effective and safe treatment for esophageal achalasia and achalasia-related diseases in patients aged 75 years and over.

## 1. Introduction

Esophageal achalasia (EA) is a disease caused by the inadequate relaxation of the lower esophageal sphincter (LES), or the impaired peristalsis of the esophagus [1,2]. The incidence rate of EA was calculated to be from 0.81 to 1.37 per 100,000 person-years, and the period prevalence rate was 7.0 per 100,000 persons. [3]. EA presents with such clinical symptoms as dysphagia, chest pain, and food regurgitation. Additionally, it progresses gradually to dysphagia and weight loss, eventually leading to a significantly decreased quality of life [2,4]. EA occurs most commonly between the ages of 20 to 60 years but sometimes occurs in the elderly. EA may cause the regurgitation of esophageal contents in elderly individuals with an impaired swallowing function, resulting in aspiration pneumonia [5]. According to Sian et al., 40% of 110 patients with EA complained of at least one respiratory symptom every day [6]. In a study of 38 patients with EA, 12 patients (31.6%) had septal thickening and necrotizing pneumonia [7]. Therefore, we should pay special attention to elderly patients who are at risk of aspiration.

Pneumatic dilation (PD) and surgical treatment, such as Heller myotomy (HM), have been used to treat EA [8,9,10,11]. However, PD is frequently ineffective while HM is invasive. Moonen et al. reported that 25% of EA patients who received PD required retreatment [9]. Thus, peroral endoscopic myotomy (POEM) was developed by Inoue in 2010 [12]. POEM is an endoscopic technique for performing a myotomy, similar to HM, but less invasive. Many studies have demonstrated the efficacy and safety of POEM [13,14,15,16]. Among them, in a multicenter prospective study in Japan, the efficacy rate of POEM was reported to be 97.4% [16]. Therefore, POEM has become the first-line treatment for EA worldwide.

However, in elderly patients with EA, there are limited reports on the efficacy of POEM [17,18,19]. In an international multicenter study of patients in their eighties with EA [19], sufficient procedural and clinical success rates of 90.8% and 93.4%, respectively, were reported. In that cohort study, 14 of 11 patients experienced adverse events (AEs) (the AE rate per patient was 14.5%). The percentages of AEs graded as mild, moderate, and severe were 78.6%, 14.3%, and 7.1%, respectively.

Therefore, the safety of POEM for elderly EA patients remains controversial. Hence, we aimed to evaluate the efficacy and safety of POEM for elderly patients, aged over 75 years, with EA or EA-related esophageal motility disorders.

## 2. Materials and Methods

### 2.1. Study Design and Study Population

We conducted a single-center retrospective observational study. We consecutively extracted patients with EA or EA-related esophageal motility disorders who underwent POEM at Fukushima Medical University Hospital, which is one of the largest hospitals in Fukushima Prefecture in Japan, between August 2014 and May 2021. Among them, the patients aged at least 75 years at the time of treatment were included in the study. Patients who were not available for any postoperative evaluation were excluded.

This study was reviewed and approved by the Ethics Committee of Fukushima Medical University (approval no. 1974). Additionally, this study was conducted in accordance with the Declaration of Helsinki of the World Medical Association. Before the procedure, all the patients provided written informed consent for POEM.

### 2.2. POEM Procedures

POEM was performed using the method reported by Inoue et al. [12]. It was performed with patients in the supine position and under general anesthesia with endotracheal intubation [20]. An endoscope with a water jet function and carbon dioxide insufflation was used. An electrosurgical Triangular-tip Knife (KD-645L; Olympus Corporation, Tokyo, Japan) with an integrated water jet function was utilized for the entire procedure to perform the mucosal incision, submucosal tunneling, and myotomy. A submucosal tunnel of approximately 1–2 cm was created through the esophagogastric junction (EGJ) to the proximal stomach. In all cases, the myotomy was performed selectively only on the circular muscle. Hemostasis was achieved with coagulation forceps (Coagrasper, FD-411QR; Olympus Corporation, Tokyo, Japan) in soft coagulation mode. The double-scope method was used to check the length and direction of the myotomy and the validity of the LES incision [21]. Once the myotomy was completed, the mucosal incision area was closed with endoclips.

The medical team for the POEM included one doctor, who was a well-experienced operator, one assistant doctor, and one clinical laboratory technician. Because the procedure was performed in the operating room, one anesthesiologist, and one nurse who managed the patient, also participated in the treatment. The procedure was estimated to last for approximately 90 min.

### 2.3. Outcomes and Definition

The primary endpoint was the clinical success rate after 2 months of POEM. Clinical success was defined as a postoperative Eckardt score of 3 or less, without the use of additional treatments. The Eckardt score was evaluated from 0 to 12 points by interviewing patients, with 12 points representing the most severe symptoms [22]. Secondary endpoints included the procedural success rate, which is defined as the completion of esophageal and gastric myotomy, the cumulative treatment effect maintenance rate, which is defined as continuous clinical success after the POEM, a change in the Eckardt score before and after POEM, the perioperative management of patients taking antithrombotic drugs, and AEs related to POEM, including gastroesophageal reflux disease (GERD) symptoms.

The degrees of esophageal dilatation were measured by the maximum diameter of the esophageal lumen on the esophagography, according to the criteria of the Japanese Esophageal Society [2], and were classified as Grade I (<3.5 cm), Grade II (3.5–6.0 cm), and Grade III (>6 cm). The types of esophageal dilatation were classified as strait, sigmoid, and advanced sigmoid. High-resolution manometry (HRM) with the Starlet system (Star Medical, Inc., Tokyo, Japan) was performed to determine the esophageal pressure. The integrated relaxation pressure (IRP) and other parameters were measured by HRM, and patients with EA were classified according to the Chicago Classification [23]. The general condition of the patients was classified according to the American Society of Anesthesiologists Physical Status (ASA-PS) Classification System [24]. POEM-related AEs were evaluated on the basis of the severity classification for AEs of the American Gastroenterological Endoscopy Society [25].

Preoperatively, and 2 months postoperatively, the patients were interviewed to obtain the Eckardt scores. Additionally, an esophagogastroduodenoscopy (EGD), esophagography, and HRM were conducted at the same time. Furthermore, the interview to obtain the Eckardt score, the evaluation for GERD symptoms, and the EGD were performed annually thereafter. The evaluation of reflux esophagitis (RE) was based on the Los Angeles classification by EGD.

### 2.4. Statistical Analysis

Measurement values were expressed as the median (range) or mean ± standard deviation (SD). Outcomes between parameters were compared before and after procedures by using the *t*-test (paired *t*-test where applicable) and the Wilcoxon signed-rank test for continuous variables. The cumulative treatment effect maintenance rate was estimated using the Kaplan–Meier method. Differences were considered statistically significant at *p* < 0.05. These analyses were performed using SPSS software version 26.0 for Windows (IBM Corp., Armonk, NY, USA).

## 3. Results

### 3.1. Patient Characteristics

**Figure 1:**
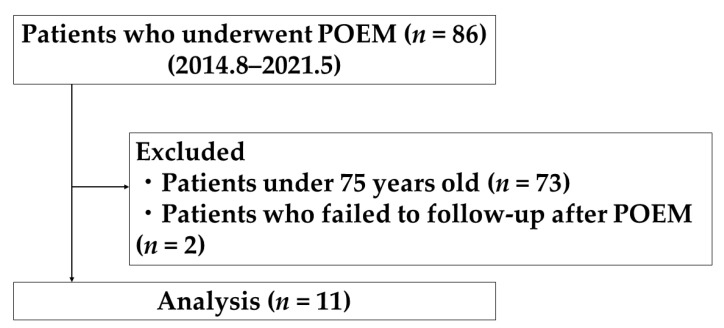
Flow diagram of patient enrollment.

A total of 86 consecutive patients with EA or EA-related esophageal motility disorders underwent POEM. Among them, 13 patients (15.1%) were at least 75 years old at the time of treatment. Finally, a total of 11 patients (12.8%) were included in this study, excluding 2 patients who could not be followed up after treatment (Figure 1).

Patient characteristics are summarized in Table 1. The median age was 81 years (range 75–87 years), and 7 patients (63.6%) were male. A total of 10 patients (90.9%) were diagnosed with EA, and 1 patient with jackhammer esophagus. A total of 4 patients, whose catheters for the HRM did not pass through the EGJ, were diagnosed with EA on the basis of the findings of the EGD and esophagography. Additionally, the median duration of symptoms was 5 years (range 2–40 years), and 5 patients (46.7%) underwent PD before POEM. The median baseline Eckardt score before the POEM was 7 (range 2–12). A total of 10 patients (90.9%) had comorbidities, and 4 patients received antithrombotic drugs.

### 3.2. Clinical Outcome and Perioperative Management of POEM

The technique of POEM is presented in Table 2. The POEM procedure was successful in all patients, including 4 patients who were taking antithrombotic drugs (Table 3). A total of 3 patients were on rivaroxaban, and 2 discontinued rivaroxaban on the day before the POEM, received heparin replacement, and restarted rivaroxaban on the day after the POEM. The other patient discontinued rivaroxaban only on the day of the POEM. One patient who received low-dose aspirin discontinued it only on the day of the POEM.

AEs other than GERD occurred for four events in 3 patients (27.3%), as described below. There was 1 patient that developed a mucosal injury without perforation, and another developed a mucosal injury without perforation and pneumoperitoneum. Mucosal injury was conservatively improved by fasting for several days, and pneumoperitoneum was gradually improved by CO_2_ absorption. Those events were specific to the POEM procedure but did not result in any issues in the clinical course. There was 1 patient that developed mild pneumonia that improved quickly with antimicrobial therapy. All AEs were classified as mild, according to the severity grading system [25]. No AEs, such as bleeding or thromboembolism, were observed.

RE occurred in 3 patients (33.3%: 2 with Grade A and 1 with Grade D), and the patient with Grade D had some symptoms that were improved by proton pump inhibitors (PPIs; Table 4).

### 3.3. Short- and Long-Term Effects of POEM

**Figure 2:**
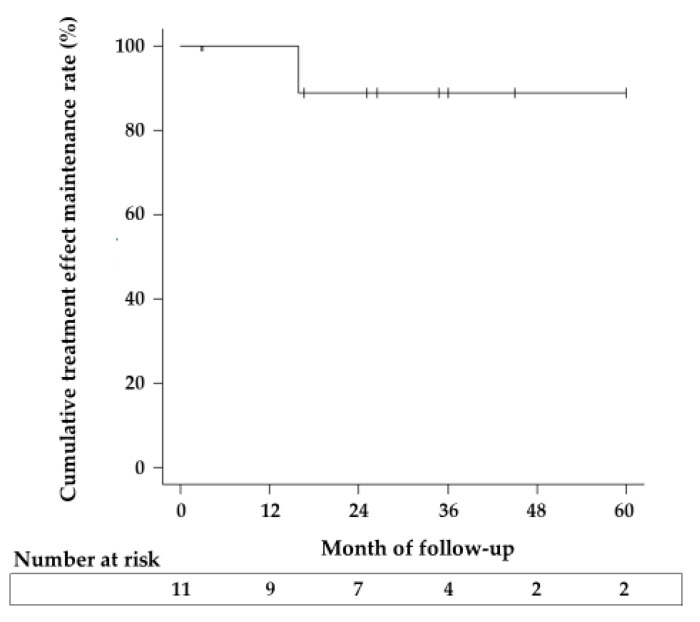
Cumulative treatment effect maintenance rate. The cumulative treatment effect maintenance rate for patients treated with POEM (peroral endoscopic myotomy) was 100% at 1 year, and 88.9% at 2 and 3 years, respectively.

The clinical success rate at 2 months after the POEM was 100%. The cumulative treatment effect maintenance rate was 100% at 1 year after POEM, and it remained at 88.9% after the second year (Figure 2). Dysphagia worsened in one patient (case 3) at 1.3 years after POEM because of the decreased treatment effect. This patient received PD that stabilized her symptoms. The Eckardt score (median [range]) was significantly lower at 2 months after POEM (0 [0,1]) than at baseline (7 [2,3,4,5,6,7,8]) (*p* < 0.01). The median Eckardt score at 1, 2, and 3 years after POEM was 0 (*n* = 9), 0.5 (*n* = 7), and 0 (*n* = 4), respectively. Additionally, the IRP values (mean ± SD) significantly decreased in seven patients who were evaluated by HRM at baseline and after POEM (baseline vs. 2 months after POEM; 26.86 ± 13.88 mmHg vs. 9.36 ± 7.93 mmHg, *p* < 0.0125).

## 4. Discussion

This study evaluated the efficacy and safety of POEM for esophageal motility disorders, mainly EA, in patients who are at least 75 years old. To the best of our knowledge, this is the first report on the long-term results of POEM in EA patients aged at least 75 years. The short-term clinical success rate of POEM in elderly patients has been reported to be 90.8–100% [17,18,19], and was 100% in this study. In the present study, the cumulative treatment effect maintenance rate after POEM was 88.9% at 3 years. We considered that POEM for EA and EA-related esophageal motility disorders in patients aged at least 75 years could have sufficient long- and short-term outcomes.

To date, the long-term outcomes of POEM in elderly patients have not been clarified. He et al. [26] examined the long-term efficacy of POEM in a relatively young population (median age: 45 years) and reported that the treatment success rate at 2 and 3 years was 90.3% and 89.0%, respectively. Similarly, Guo et al. [27] reported that the long-term outcome (with a minimum follow-up of 3 years) of POEM in a young population (mean age: 40.7 years) was 88.1%. The results of the present study are consistent with previous reports, indicating that POEM could ensure long-term efficacy in elderly patients. Moreover, the efficacy of POEM in elderly patients might be discussed in the same manner as for younger patients.

In this study, 46.7% of the patients had a history of PD. In the cases of laparoscopic HM for EA, fibrosis of the submucosa caused by the previous PD reduced the efficacy of the treatment [28]. There is a concern that a history of PD may also reduce the efficacy of POEM [29]. Chen et al. [19] reported that one of the causes of the discontinuation of POEM in patients over 80 years of age was fibrosis of the submucosa due to previous treatment or long disease duration. It was also reported that creating a submucosal tunnel was difficult when the esophagus was markedly dilated or tortuous [17,19,30]. In the present study, 46.7% of patients had a history of PD, and 2 patients had sigmoid achalasia with a disease duration of 20 and 40 years. However, all patients underwent POEM, and their symptoms improved. POEM could contribute to the improvement of symptoms in elderly patients with a history of PD and sigmoid EA if the careful creation of a straight submucosal tunnel to the gastric side without mucosal injuries is possible.

It has been demonstrated that esophageal motility and anatomical changes are associated with aging, and the concept of presbyesophagus is well-known [19,31]. In this study, of the six cases of EA that could be evaluated by HRM, four (66.7%) were Chicago Classification Type I and two (33.3%) were Type II. This could be attributable to the difference in case numbers, though the proportion of Type II cases was larger in previous reports [18,19]. In esophagography, the sigmoid type was the most common (72.7%), which was higher than that of previous reports (31.5%) [19]. Conversely, Grade I esophageal dilatation was observed in 63.6% of our cases. However, we evaluated only a small number of cases herein. The characteristics of HRM and the findings of esophagography in elderly patients with achalasia should be further investigated with a large number of cases.

Because POEM is performed under general anesthesia with tracheal intubation, we should consider the decline in the physical and physiological functions associated with aging in elderly patients [32]. In this study, 90.9% of the patients had ASA-PS class II, but none of them developed circulatory disorders during or after POEM. Only one patient had pneumonia, which improved after conservative treatment.

In terms of AEs, three patients (27.3%) had AEs in this study. However, no serious or fatal AEs were observed. In a study of patients over the age of 80 years [19], the incidence of AEs was 14.5%. By comparison, the incidence of AEs in this study appears to be higher. This may be due to the small number of cases. Furthermore, this study differs in that there were no moderate or severe AEs. Here, AEs were observed in 20 (26.7%) of 75 young patients who underwent the procedure during the same period. The AEs included pneumoperitoneum without a puncture (11 cases), pneumoperitoneum with a puncture (5 cases), a mucosal injury without perforation (3 cases), and separation of the entry site (1 case). There was 1 case with a separated entry site classified as severe, but all other cases were mild and improved with conservative treatment. The incidence of AEs was compared between elderly and young patients, but no significant difference was observed (*p* = 1.0) (data not shown). Although the incidence of AEs in elderly patients may be the same as that in younger patients, it is important to note that there is a combination of factors involved, such as the age-related decline in the physiological reserve, and age-related changes in the esophageal tissue [19].

The incidence of postoperative bleeding for POEM has been reported to be significantly higher in patients taking antithrombotic drugs [33]. In the present study, four patients (36.4%) received antithrombotic medications, but no hemorrhage or thromboembolism was observed. Apart from that, mild AEs related to POEM were observed in three patients (27.3%) but improved without serious difficulties.

The incidence of GERD after POEM in the elderly has been reported to be 6.7% to 16.1% [17,18,19]. In our study, symptomatic GERD after POEM occurred in only one case (9.1%) and was successfully treated with PPIs. Although GERD may occur after POEM, symptoms improve with PPIs in most cases [15,17,18]. There are several reports that symptomatic GERD after POEM can be controlled with PPIs, and it is difficult to speculate that elderly patients are particularly poorly controlled. Therefore, further studies are needed.

This study has several limitations. First, it is a retrospective study of a small number of patients at a single institution. However, there is little selection bias because of the consecutive cases. Second, there were no Type III EA cases as defined by the Chicago Classification. Third, POEM was the only treatment, and there were no comparisons with other treatments. Fourth, there is a selection bias in that only the patients who were able to choose POEM as a treatment were included in the study.

## 5. Conclusions

In this study, we showed that POEM could be safely performed in patients aged at least 75 years with EA and EA-related esophageal motility disorders. The treatment effect was sufficient for up to 3 years. POEM could be the first-line option for EA, even in cases of elderly patients. The long-term prognosis of the elderly with EA after POEM needs to be evaluated in a large number of patients in a multicenter setting.

## Figures and Tables

**Table 1:** Patient characteristics.

Case	Age	Sex	BMI (kg/m^2^)	ASA-PS	Diagnosis	Chicago Classification	Duration of Symptoms, (Years)	Dilatation Grade	Esophago Graphy	Previous Treatment	Eckardt Score		Comorbidity
											Baseline	2 Months after POEM	
1	77	M	26.7	II	Achalasia	Type I	7	I	Sg	None	8	1	Af, AP
2	85	F	19.8	II	Achalasia	N/A	6	II	Sg	PD	2	0	HT
3	81	F	24.0	II	Achalasia	Type I	5	II	Sg	PD	7	1	HT, CRF
4	75	M	24.8	II	Achalasia	Type II	2	I	Sg	None	6	0	HT
5	87	M	24.8	II	Achalasia	N/A	40	II	Sg	PD	7	1	Af, CRF
6	85	F	25.3	II	Achalasia	Type I	6	II	Sg	None	5	0	HT, Lacunar infarction
7	77	M	23.6	II	Achalasia	Type II	2	I	St	None	5	1	Dyslipidemia
8	86	F	28.6	II	Achalasia	N/A	3	I	St	PD	7	0	CSA, SAS
9	75	M	21.2	III	JE	JE	5	I	St	PD	8	0	Af, CHF
10	76	M	18.5	II	Achalasia	N/A	20	I	Sg	None	5	0	None
11	83	M	21.5	II	Achalasia	Type I	3	I	St	None	8	0	DM, CRF, OMI

BMI: body mass index (calculated as weight in kilograms divided by height in meters squared); ASA-PS: American Society of Anesthesiology physical status; POEM: peroral endoscopic myotomy; M: male; F: female; JE: jackhammer esophagus; N/A: not applicable. In Cases 2, 5, 8, and 10, the catheter for high-resolution manometry could not pass through the esophagogastric junction; hence, the integrated relaxation pressure could not be measured. Sg: sigmoid type; St: straight type; PD: pneumatic dilation; Af: atrial fibrillation; AP: angina pectoris; HT: hypertension; CRF: chronic renal failure; CAS: coronary spastic angina; SAS: sleep apnea syndrome; CHF: chronic heart failure; DM: diabetes mellitus; OMI: old myocardial infarction.

**Table 2:** Clinical outcomes of POEM.

Clinical success, *n* (%)		11 (100)
Procedure time *, min		109 (62–144)
Direction of myotomy	Posterior side	11 (100)
Myotomy length *, cm	Total	13 (8–19)
	Esophageal side	10 (5–16)
	Gastric side	3 (2–3)
Eckardt score 2 months after POEM *		0 (0–1)
Adverse events ^†^, *n* (%)	Total	3 (27.3)
	Pneumoperitoneum	2 (18.2)
	Mucosal injury without perforation	1 (9.1)
	Pneumonia	1 (9.1)

* Data are shown as median (range). ^†^ In one case, both pneumoperitoneum and mucosal injury were seen. POEM: peroral endoscopic myotomy.

**Table 3:** Perioperative management of the patients taking antithrombotic drugs.

Case	Antithrombotic Agents	Perioperative Management	Antithrombotic Resumption	Adverse Events
1	Rivaroxaban	Discontinued on the day before POEM with heparin bridging	Next day	None
5	Rivaroxaban	Discontinued on the day before POEM with heparin bridging	Next day	None
6	Aspirin	Discontinued on the day before POEM	Next day	None
9	Rivaroxaban	Discontinued on the day before POEM	Next day	None

Af: atrial fibrillation; AP: angina pectoris; CHF: chronic heart failure; DM: diabetes mellitus; OMI: old myocardial infarction; POEM: peroral endoscopic myotomy.

**Table 4:** GERD after POEM.

Reflux Esophagitis, *n* (%)	
Grade A	2 (18.2)
Grade D	1 (9)
Symptomatic GERD, *n* (%)	1 (9)

GERD: gastroesophageal reflux disease; POEM: peroral endoscopic myotomy.

## Data Availability

Data available upon request because of restrictions, e.g., privacy or ethical concerns.
